# Use of noninvasive mechanical ventilation weaning protocol in neonatal intensive care units in Brazil: a descriptive study

**DOI:** 10.1590/1984-0462/2023/41/2021382

**Published:** 2023-05-15

**Authors:** Jéssica Delamuta Vitti, Antonio Adolfo Mattos de Castro, Nelson Francisco Serrão

**Affiliations:** a Instituto Educacional Campos, Campinas, SP, Brazil.; b Universidade Federal do Pampa, Uruguaiana, RS, Brazil.

**Keywords:** Noninvasive ventilation, Ventilator weaning, Intensive care units, neonatal, Infant, premature, Continuous positive airway pressure, Brazil, Ventilação não invasiva, Desmame do respirador, Unidades de terapia intensiva neonatal, Recém-nascido prematuro, Pressão positiva contínua nas vias aéreas, Brasil

## Abstract

**Objective:**

This study aimed to investigate whether neonatal intensive care units (NICUs) in Brazilian hospitals use a protocol for weaning from noninvasive ventilation (NIV), how this ventilatory support is withdrawn, and whether there is consensus among the methods used by the institutions.

**Methods:**

A cross-sectional survey was conducted from December 2020 to February 2021, based on responses to an electronic questionnaire, filled out by physical therapists working in NICU in Brazilian hospitals about the routine of physical therapy and the use of NIV and its weaning.

**Results:**

A total of 93 answers to the electronic questionnaire met the study criteria: 52.7% were from public health institutions, with an average of 15 NICU beds (15.2±15.9), 85% of the physical therapists worked exclusively in the NICU, 34.4% of the NICU had 24-h physical therapy care, 66.7% of the units use the continuous positive airway pressure (CPAP) as ventilatory mode, and 72% the nasal prong as NIV interface; 90% of the NICU physical therapists answered that their NICU had no NIV weaning protocol, with various methods of weaning reported, the most cited being pressure weaning.

**Conclusions:**

Most Brazilian NICUs have no NIV weaning protocol. The most used method among institutions, with or without a protocol, is pressure weaning. Although most of the participating physical therapists work exclusively in NICU, many hospitals do not have the recommended workload, which can be one of the negative factors in the organization of protocols and in the progress of ventilatory weaning.

## RESUMO

**Objetivo:**

Investigar se as unidades de terapia intensiva neonatal (UTIN) dos hospitais brasileiros utilizam protocolo para desmame de ventilação não invasiva (VNI), de que forma é realizada a retirada desse suporte ventilatório e se há consenso entre os métodos utilizados pelas instituições.

**Métodos:**

Foi realizado um estudo de corte transversal de dezembro de 2020 a fevereiro de 2021, com base nas respostas a um questionário eletrônico, preenchidas por fisioterapeutas que trabalham em UTIN de hospitais brasileiros sobre a rotina da fisioterapia, o uso de VNI e seu desmame.

**Resultados:**

Preencheram os critérios do estudo 93 respostas ao questionário eletrônico: 52,7% foram de instituições públicas de saúde, com média de 15 leitos de UTIN (15,2 ±15,9); 85% dos fisioterapeutas trabalhavam exclusivamente na UTIN, 34,4% das UTIN possuíam atendimento fisioterapêutico 24 horas por dia; 66,7% das unidades utilizam o Continuous Positive Airway Pressure (CPAP) como modo ventilatório e 72% a pronga nasal como interface de VNI; 90,3% dos fisioterapeutas responderam que suas UTIN não possuíam protocolo de desmame de VNI, com métodos de desmame relatados variados, sendo mais citado o desmame de pressão.

**Conclusões:**

A maior parte das UTIN brasileiras não possui protocolo de desmame de VNI; o método mais utilizado entre as instituições que possuem ou não protocolo é o desmame de pressão. Apesar de a maioria dos voluntários trabalhar exclusivamente em UTIN, muitos hospitais não possuem a carga horária recomendada, o que pode ser um dos fatores negativos na organização dos protocolos e no andamento do desmame ventilatório.

## INTRODUCTION

Neonatal intensive care units (NICUs) are intended for hospital care of patients between 0 and 28 days of life,^1^ and neonatal hospitalization is mainly related to lower gestational age (GA), low birth weight, and need for oxygen therapy or ventilatory support, which are associated with higher neonatal morbidity and mortality.^2-4^

Respiratory difficulty in this population occurs due to pulmonary and respiratory center immaturity, added to the anatomical disadvantages of the conduction airways, predisposing to asynchronous respiratory movements, respiratory pauses, and apneas.^5^

Noninvasive ventilation (NIV) provides positive pressure to the airway via mask or nasal prong, acting to prevent and treat patients with respiratory distress (RD). The ventilatory mode and its parameters must be chosen according to the age, weight, clinical picture, and individual tolerance of each patient. Its benefits include alveolar recruitment, prevention of airway collapse and obstructions, improved oxygenation and decreased ventilatory workload, and reduced infection rates when compared to invasive mechanical ventilation.^6-8^

Weaning from NIV is the process of withdrawing ventilatory support and can be initiated when there is hemodynamic stability, improvement in the RD, and absence of significant respiratory pauses. The literature^9-17^ presents various ways of performing this process, which can occur abruptly or in intervals, with progressive reduction of ventilatory pressures or increased time without ventilatory support, use of oxygen therapy, and high-flow nasal cannula (HFNC), among others.

Although there are studies^9-17^ on weaning from NIV, their findings are controversial, and few address weaning protocols. Furthermore, there is a scarcity of studies presenting the reality of weaning in Brazilian hospitals, and it is not possible to state the start and interruption criteria, RD, current weight, corrected GA and the need for oxygen, as well as the ideal moment, since early withdrawal of this ventilatory support may promote atelectasis, apnea, bradycardia, and, consequently, a decrease in oxygen therapy, ventilatory modes, and parameters. Likewise, prolonging ventilatory support may increase the incidence of septal injury, retinopathy of prematurity, nosocomial infections, and length of hospital stay.

The method and optimal timing for weaning from NIV varies and ultimately depends on the subjective clinical judgment of each team.^9,10^ Therefore, this study investigated whether NICU in Brazilian hospitals used a protocol for weaning from NIV, how this ventilatory support was withdrawn, and whether there was a consensus between the methods used among the institutions.

## METHOD

A cross-sectional study was conducted from December 2020 to February 2021, based on information obtained from an electronic form (*Google form*) by physical therapists working in Brazilian hospitals with NICUs and performing NIV in patients admitted to this sector.

Responses to the form by physical therapists working in the same institution were excluded. Only the first response received from each institution was considered, discarding later responses. Only one of the authors had access to the form during data collection, and the other authors were blinded to the exclusion of duplicate responses and identification of participants.

The Ethics and Research Committee of the Universidade Federal do Pampa approved this study (no. 4,341,613). The Written Informed Consent Form (WIFC) was made in digital form, together with the electronic form.

Physical therapists working in NICU were contacted via *email*, telephone, social networks (e.g., Facebook, WhatsApp, Instagram, and LinkedIn), and other contact networks, through which they received the link to access the electronic form and were invited to participate in this study. This research was continued in the “snowball” format, where each professional could send the access link of the electronic form to other physiotherapists working in NICU.

The electronic form was composed of 12 questions, of which 1 was the WIFC; 6 were related to routine, physiotherapy, and NIV at the institution; 2 were about NIV protocol and weaning; and 3 were about volunteers’ identification and questions. The form allowed three types of answers: objective, open numerical, and open written, according to the question asked. The electronic form data is detailed in Table 1.

**Table 1.** The questions and answer options found in the electronic survey form.

n^o^	Questions	Answer options
1	Does the hospital where you work provide public or private care?	Public
		Private
		Both
2	How many beds does the NICU at the hospital where you work have?	(open numeric response)
3	Is there an exclusive physiotherapist for this sector?	Yes
		No
4	What is the workload of physiotherapy in this unit?	<6 h
		6 h
		8 h
		12 h
		18 h
		24 h
5	What is the most commonly used mode of noninvasive ventilation in the sector?	CPAP
		BIPAP “noninvasive ventilation”
		Others
6	What types of interface are used on your unit?	Nasal prong
		Nasal mask
		Prong and nasal mask
		No interface
		Others
7	Does the institution where you work have a protocol for weaning from noninvasive ventilation?	Yes
		No
8A	If “yes,” describe or send the protocol.	(open response dissertative)
8B	If “no,” describe how weaning from noninvasive ventilation is performed in your institution	(open response dissertative)
9	Institution name	(open response dissertative)
10	Contact email	(open response dissertative)
11	Comments and/or doubts	(open response dissertative)

NICU: neonatal intensive care unit; CPAP: continuous airway pressure; BIPAP: ventilatory mode with two airway pressures.

The sample size was calculated from the total number of Brazilian hospitals with qualified NICU, according to the National Registry of Health Establishments of the Ministry of Health,^18^ totaling a total national sample of 450 NICUs. The sample size calculation considered a significance of α=0.05 and a statistical power of 1−β=0.95, resulting in a minimum sample size of 90 Brazilian NICUs, and therefore, 90 responses to the electronic form.

The data were analyzed according to the types of answers (i.e., objective, numerical open, and written open). For the objective answers, the chi-square method was used to analyze the proportion of answers; for the objective and numerical open answers, the Student’s t-test was used, with p-value <0.05; and for the open answers, a descriptive and comparative analysis was carried out based on literature data:^10,11,13,15-17^ criteria for initiation, interruption, success, and failure of ventilatory weaning, as well as GA, weight, and O_2_ use.

## RESULTS

There were 114 responses to the electronic form. A total of 109 of the participating physiotherapists answered “yes” to the informed consent form and were guided to the specific research questions. Among those who answered “yes,” 16 esponses identified as duplicate institution were excluded. Finally, 93 responses to the electronic form were included in this study, made by physical therapists working in NICU from different hospital institutions, totaling 22 states, covering the 5 Brazilian regions.

From the 93 responses analyzed, 52.7% of the NICUs were part of public health institutions. The institutions had an average of 15 NICU beds (15.2±16), but the number of beds varied considerably among the responses, with a minimum reported value of 6 and a maximum of 120 beds.

In relation to physiotherapy in the NICUs, 85% had a physical therapist exclusively for the sector, as for the workload, 34.4% of the NICUs had physiotherapy care 24 ha day, 21.5% had 18 h care, 19.4% had 12 h care, and the others had 8, 6, and <6 h of physiotherapy in the sector (3.2, 13.6, and 7.5%, respectively).

The most commonly used NIV mode in NICU was continuous positive airway pressure (CPAP) (66.7%), followed by bilevel positive airway pressure (BIPAP) (25.8%). As for the interface used, 72% of the NICUs used only nasal prongs; 25.8% of the NICUs used a prong and a nasal mask, and only 2.2% of the NICUs used only the nasal mask as an interface for NIV. No other interface was informed by the volunteers, and 100% of the institutions used at least one of the interfaces mentioned above.

Regarding the NIV weaning protocol, 9 (9.7%) of the volunteer physical therapists reported that the NICU where they worked had a protocol and 84 (90.3%) reported that they did not have a protocol for NIV weaning.

**Figure 1.**
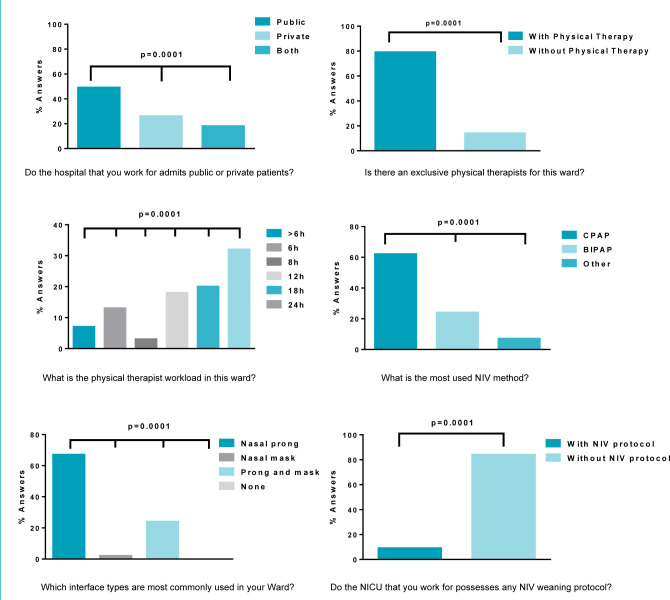
Proportions between objective answers from the electronic form.

NICU: neonatal intensive care unit; CPAP: continuous positive airway pressure; BIPAP: two-level positive airway pressure; other: other ventilatory mode; none: no interface; NIV: noninvasive ventilation.

Figure 1 replicates the proportions between the objective answers to the electronic form as “yes” and “no,” understanding that when answering “yes” to one alternative, the volunteer answered “no” to the others: obtaining a higher proportion of “yes” answers for public hospitals (85%), 24 h physiotherapeutic assistance (66.7%), CPAP use (66.7%), nasal prong (72%), and absence of NIV weaning protocol (90.3%). The p-value obtained in the evaluated data was extremely significant (p<0.001) in all objective questions, validating the data used in this research (Table 2; Figure 1).

**Table 2.** Survey form questions and answers.

Questions	Answers
1. Does the hospital where you work provide public or private care?	(n/%)
Public (attends patients from the Unified Health System – SUS)	(49/52.68%)
Private (serves private and health plan patients)	(26/27.96%)
Both (serves SUS, health plan, and private patients)	(18/9.35%)
Total	(93/100%)
2. How many beds in the NICU of the hospital where you work?	(N/M±SD)
(numerical open answer)*	(1410/15.16±15.92)
3. Is there an exclusive physiotherapist for this sector?	(n/%)
Yes	(79/84.95%)
No	(14/15.05%)
Total	(93/100%)
4. What is the workload of physiotherapy in this unit?	(n/%)
<6 h	(7/7.53%)
6 h	(13/13.98%)
8 h	(3/3.23%)
12 h	(18/19.35%)
18 h	(20/21.50%)
24 h	(32/34.41%)
Total	(93/100%)
5. Which noninvasive ventilation mode is most used in the sector?	(n/%)
CPAP	(62/66.67%)
BIPAP	(24/25.80%)
Other	(7/7.53%)
Total	(93/100%)
6. Which types of interface are used in your unit	(n/%)
Nasal prong	(67/72.04%)
Nasal mask	(2/2.15%)
Prong and nasal mask	(24/25.81%)
No interface	(0/0%)
Other	(0/0%)
Total	(93/100%)
7. Does the institution where you work have a protocol for weaning from noninvasive ventilation?	(n/%)
Yes	(9/9.68%)
No	(84/90.32%)
Total	(93/100%)

NICU: neonatal intensive care unit; M: mean; SD: standard deviation; NA: not applicable; CPAP: continuous positive airway pressure; BIPAP: two levels of positive airway pressure. *This question accepted numerical answers freely filled in by the volunteer.

**Figure 2.**
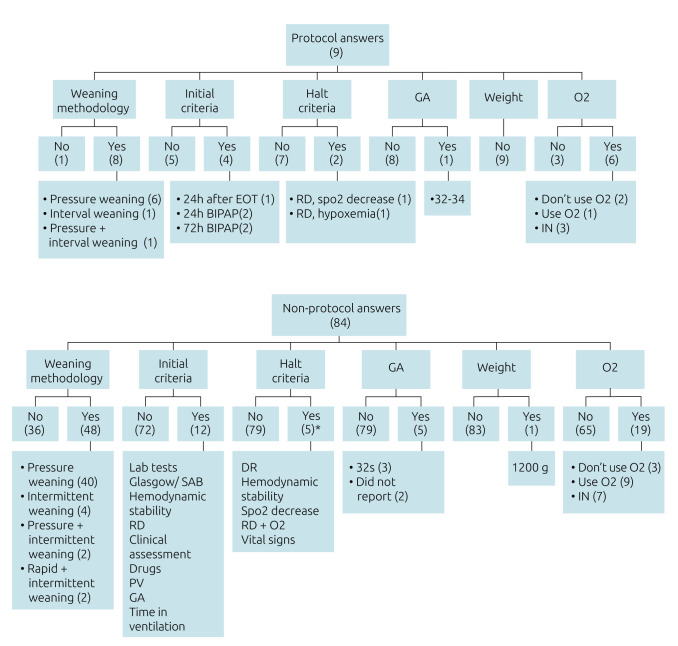
Criteria for evaluation and analysis of the essay answers (R) of questions 8A and 8B of the electronic form.

GA: gestational age; no=answer given by the volunteer did not allow identification of this assessment criterion; yes: answer given by the volunteer allowed identification of this assessment criterion; EOT: extubation; BIPAP: positive airway pressure at two levels; SPO2: peripheral oxygen saturation; IN: if needed; SAB: Silverman-Andersen Bulletin; RD: respiratory distress; PV: ventilatory parameters; s: weeks of gestation. *Interpretation of the answers given by the physiotherapists with the same meaning.

The open-ended written responses related to weaning from NIV were analyzed in terms of weaning methodology, use of criteria for initiation and interruption of weaning, oxygen therapy, GA, and minimum weight. The most reported weaning method among the participants in this study was pressure weaning (49.5%), both among those who reported having a protocol at their NICU and those who reported no protocol (Figure 2).

Analyzing separately and grouping the answers into having a protocol (answers to question 8A of the electronic form) and having no protocol (answers to question 8B of the electronic form); in the answers with a protocol, the most reported weaning method was pressure weaning with 6 (66.7%) answers. One physical therapist answered *consensus protocol*, without further specification, not allowing the identification and inference of the variables analyzed at this stage.

Notably, 4 (44.4%) protocol responses identified criteria for initiating weaning and 2 (22.2%) identified criteria for stopping weaning. Only 1 (11.1%) protocol response considered the patient’s GA but none of them considered the patient’s weight. As for O_2_, 6 (66.7%) physical therapists specified its use outside the ventilatory support: 1 (11.1%) physical therapist used O_2_ in the interval periods of ventilatory support, 2 (22.2%) physical therapists did not use O_2_ in any stage of weaning, 1 (11.1%) physical therapist returned the patient to NIV in case of a drop in peripheral oxygen saturation (SPO_2_), and 1 (11.1%) physical therapist used O_2_ only when it was necessary, being placed in room air first. The other physical therapists (3, 33.3%) did not specify about O_2_ use.

In the answers without a protocol, the most used method of weaning was pressure weaning with 40 (47.6%) answers, but other methods were also reported: interval weaning (4.8%), abrupt weaning (2.4%), and pressure weaning followed by interval weaning (if the abrupt weaning failed, interval weaning was performed) (2.4%), and in 36 (42.9%) answers, it was not possible to identify the weaning methodology used at the institution.

In addition, 12 (14.3%) non-protocol responses reported criteria for initiating weaning, 5 (6%) responses reported stopping criteria for weaning, and 5 (6%) physical therapists responded that GA influenced weaning. Regarding the use of oxygen therapy without ventilatory support, 19 were obtained (22.7%) answers: 9 (10.7%) physical therapists used O_2_ in the weaning process, 3 (3.6%) did not use O_2_, and 7 (8.3%) used O_2_ only when necessary.

In the responses without protocol, other variables that interfered in the process of weaning from NIV were identified: variation of the weaning method according to the on-call physician or other professionals (11.9%), use of complementary tests (17.9%) and clinical assessment scales in the weaning decision process (2.4%), permanence of the ductus arteriosus as a negative factor in the weaning process (1.2%), correct size of the prong, use of hydrocolloid and non-pharmacological pain measures as a measure of weaning success (1.2%), and individual discussion of the cases by a multiprofessional team, justifying the absence of a protocol (1.2%).

## DISCUSSION

The volunteer physical therapists in this study answered the form that most of them worked in public health institutions, performed physiotherapy exclusively in NICUs, in institutions with less than 24 h of physiotherapy care, used predominantly CPAP mode, nasal prongs, and had no protocol for weaning from NIV. Regardless of protocol use, the pressure weaning method was the most commonly reported in this research.

In the literature,^9,15^ several methods of weaning from NIV are found, the most commonly reported being pressure weaning, interval weaning, and abrupt weaning, corroborating the findings of this study. Although these methods are the most cited, there are several ways to perform them, varying according to the minimum pressure used for weaning, patient’s conditions at the beginning of weaning, positioning in bed, minimum and maximum time of weaning, interface used, skin care, oxygen therapy during and after weaning, as well as some criteria that can be determined to start and stop the weaning process.^10^

The criteria for initiating weaning vary among authors^11,13,15,16^ and include variables such as minimum GA, minimum pressure level after extubation, delimited O_2_ with no increase in the last 12–24 h, absence of RD, apnea, bradycardia, SPO_2_ drop, hemodynamic stability, adequate gasometry, use of caffeine, not treating patent ductus arteriosus or sepsis, and tolerating CPAP withdrawal during routine care (15 min).

The criteria for failure of weaning from NIV, as well as those for initiation, showed a failure rate of more than 50%, suggesting that the criteria used were insufficient to identify whether neonates were suitable for weaning.^19^ These varied according to the weaning methodology performed and suggest that weaning should be stopped in cases of marked RD, tachypnea, need for increased O_2_, gasometric change, apnea or bradycardia requiring resuscitation, manual resuscitator ventilation, or intubation.^13,16,17^

In case of failure, ventilatory support (CPAP) was returned for 24 h until stability criteria were achieved again.^14^ To be considered successful in weaning from NIV, the newborns should remain 24 h or 7 days without ventilatory support, with absence of RD, tachypnea, apnea or bradycardia, in room air, or with sufficient O_2_ supplementation to maintain adequate SPO_2._^17^ Factors such as previous intubation, anemia, infection, and gastroesophageal reflux could influence the success of weaning and would be associated with a longer weaning time.^17^ The start and failure criteria answered by the physiotherapy in this research corroborate those found in the literature, as well as the absence of a consensus on its performance.

The use of O_2_ in the process of weaning from NIV is still controversial, as the help of oxygen therapy in early weaning solves hypoxemia but does not overcome the physiological mechanisms of respiratory failure, which may be a masking factor for weaning failure.^10^ The use of O_2_ catheter or HFNC, when compared to CPAP, allows more comfort for the neonates, freedom of movement, and interaction with parents and caregivers, contributing to their development;^20^ on the contrary, patients who were weaned from O_2_ had a higher risk of developing bronchodysplasia and retinopathy of prematurity. Moreover, the length of hospital stay was similar in patients who weaned from CPAP to room air and to O_2_, and the use of O_2_ was not superior to other weaning methods, and patients could be weaned directly to room air.^12^ The absence of defined criteria for weaning oxygen therapy corroborates the physical therapists responses to this survey.

Nasef et al.^10^ indicated the use of nasal intermittent positive pressure ventilation (NIPPV) in situations of apnea and increased work of breathing and that the use of NIPPV in other cases in an unjustified manner could promote additional lung injury. They also state that HFNC does not provide reliable pressure necessary to stimulate lung growth and that the use of oxygen therapy masks respiratory failure, is not superior to CPAP, and should only be used in individuals in whom CPAP cannot be applied (nasal injury and home support, for example).

Kidszun et al.^20^ conducted a study, which investigated the practical care of CPAP weaning in premature infants in Germany, using an electronic form. Most of the volunteers answered that they do not use a weaning protocol (66/83–80%), corroborating the findings of this research, but the most used weaning method in German NICUs is the pressure weaning followed by the interval method, which refutes the findings of this research, reported by the physical therapists of Brazilian NICUs. O_2_ was used in cases of dyspnea, apneas, and bradycardias, and 33% of all German tertiary units used HFNC at some point during the process, justified by the priority on comfort and bonding with the parents, when compared to economic aspects. They also reported that criteria for weaning from NIV are highly variable and based on individual assessments and preferences, corroborating the findings of this study.

Tume et al.,^21^ in research on weaning in 65 pediatric intensive care units, via electronic form, in 19 European countries, pointed out that guidelines and protocols for invasive and noninvasive mechanical ventilation and weaning are not commonly used in the pediatric population, justified by the scarcity practical scientific evidence in the literature and equipment that perform this process in an automated way, unlike the adult population.

The studies by Kidszun et al.^20^ and Tume et al.,^21^ with methodologies similar to this research, corroborate the data presented and show that the absence of mechanical ventilation protocols in neonatology and pediatrics is a reality that is not limited to Brazil.

Regarding Brazilian studies, Rodrigues et al.^22^ reported that uninterrupted physiotherapy service in intensive care (24 h) reduces the time of mechanical ventilation and stay in these sectors, as well as costs, when compared to sectors with physiotherapy only 12 h a day. The authors also stated that having an exclusive physiotherapist in the sector, uninterruptedly, is directly related to the use of protocols in mechanical ventilation.

Due to the variability in practices and experiences used by healthcare professionals, neonates using NIV have varying clinical responses and outcomes. Standardization of practice and the inclusion of some precautions are essential for successful weaning, such as the use of NIV in the delivery room, checklists, escalation of care, frequent clinical evaluation, establishment of weaning initiation/interruption/success criteria, and others. The multidisciplinary team working in sync, committed, and trained keeps patients comfortable and reduces the incidence of complications, which can be a factor of success or failure according to the work environment.^9,10^

The integration of professionals from the different professions involved in intensive care is essential for cooperation and knowledge exchange, providing more adequate care to the patient, especially in urgent and emergency situations.^23^ The insertion and performance of the physical therapist is based on the current legislation,^1^ determining a minimum of 18 h of assistance in intensive care, with a limit of 10 beds per professional, and should not be limited by bureaucratic barriers or professionals who do not recognize their conduct. Although the legislation supports, recommends, and regulates the presence of physical therapist in NICU, the actual workload, routines, and techniques used in care at the municipal, state, and national levels are little known. This shortage of evidence instigates the bias toward inadequacy and devaluation of the profession, as well as the care provided to newborn infant.^23^

The limitations of this research were related to the information answered on the electronic form, which could be limited and generate self-report misunderstandings, confusion, lack of generalization, and no means of verifying the participants’ data. It was also not possible to cover all the Brazilian states, and information about the states of Alagoas, Rondônia, Roraima, Sergipe, and Tocantins were absent in this study.

From the data obtained in this research, it was possible to verify that most Brazilian NICU do not have a protocol for weaning from NIV, with pressure weaning being the most reported among the physical therapists, regardless of the use or not of a protocol; however, many did not identify the methodology and criteria used to determine the beginning, interruption, failure, and success of weaning, not being able to state a consensus on weaning practices.

Standardization of care and the use of a protocol in weaning from NIV allow for a safer process and can influence the success of weaning, as can routines in NICU and multiprofessional care. Physiotherapy is a new and growing profession; although most of the participating physical therapists work exclusively in NICU, many hospitals do not have the recommended workload, which can be one of the negative factors in the organization of protocols and in the progress of ventilatory weaning.

## Data Availability

The database that originated the article is available with the corresponding author.
